# 3,3′-Bis(3-methoxy­benz­yl)-1,1′-ethyl­enediimidazolium dibromide

**DOI:** 10.1107/S1600536808031863

**Published:** 2008-10-09

**Authors:** Hon Man Lee, Chi-Ying Lu

**Affiliations:** a National Changhua University of Education, Department of Chemistry, Changhua 50058, Taiwan

## Abstract

In the title compound, C_24_H_28_N_4_O_2_^2+^·2Br^−^, the imidazolium cation is located on an inversion centre. The two imidazole rings are parallel to each other, whereas the imidazole and benzene rings make a dihedral angle of 77.25 (16)°. Non­classical inter­molecular C—H⋯Br hydrogen bonds link the imidazolium cations and the bromide anions into a three-dimensional network.

## Related literature

For the structure of 1,1′-bis­(3-methoxy­benz­yl)-3,3′-methyl­enediimidazolium dibromide, see: Lee & Chiu (2004 ▶). For the structures of other related bis­(imidazolium) salts, see: Cheng *et al.* (2006 ▶); Lee *et al.* (2004 ▶, 2007 ▶). For a review of *N*-heterocyclic carbenes, see: Hillier *et al.* (2002 ▶).
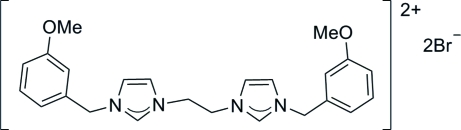


## Experimental

### 

#### Crystal data

C_24_H_28_N_4_O_2_^2+^·2Br^−^
*M* *_r_* = 564.32
Monoclinic, 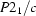

*a* = 18.340 (6) Å
*b* = 5.3566 (17) Å
*c* = 12.340 (4) Å
β = 91.491 (9)°
*V* = 1211.9 (7) Å^3^
*Z* = 2
Mo *K*α radiation
μ = 3.37 mm^−1^
*T* = 298 (2) K
0.35 × 0.20 × 0.15 mm

#### Data collection

Bruker SMART APEXII diffractometer
Absorption correction: multi-scan (*SADABS*; Sheldrick, 2003 ▶) *T*_min_ = 0.366, *T*_max_ = 0.600
6855 measured reflections
2609 independent reflections
1838 reflections with *I* > 2σ
*R*_int_ = 0.050

#### Refinement

*R*[*F* ^2^ > 2σ(*F* ^2^)] = 0.052
*wR*(*F* ^2^) = 0.143
*S* = 1.00
2609 reflections
145 parameters
H-atom parameters constrained
Δρ_max_ = 0.71 e Å^−3^
Δρ_min_ = −1.24 e Å^−3^

### 

Data collection: *APEX2* (Bruker, 2004 ▶); cell refinement: *APEX2*; data reduction: *SAINT* (Bruker, 2004 ▶); program(s) used to solve structure: *SHELXTL* (Sheldrick, 2008 ▶); program(s) used to refine structure: *SHELXTL*; molecular graphics: *SHELXTL*; software used to prepare material for publication: *SHELXTL*.

## Supplementary Material

Crystal structure: contains datablocks I, global. DOI: 10.1107/S1600536808031863/wn2283sup1.cif

Structure factors: contains datablocks I. DOI: 10.1107/S1600536808031863/wn2283Isup2.hkl

Additional supplementary materials:  crystallographic information; 3D view; checkCIF report

## Figures and Tables

**Table 1:** Hydrogen-bond geometry (Å, °)

*D*—H⋯*A*	*D*—H	H⋯*A*	*D*⋯*A*	*D*—H⋯*A*
C2—H2*A*⋯Br1^i^	0.93	2.77	3.657 (4)	161
C3—H3*A*⋯Br1^ii^	0.93	2.91	3.729 (4)	148
C4—H4*B*⋯Br1^iii^	0.97	2.85	3.669 (4)	143

Symmetry codes: (i) 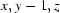; (ii) 
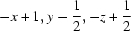
; (iii) 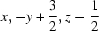.

**Fig. 1.**
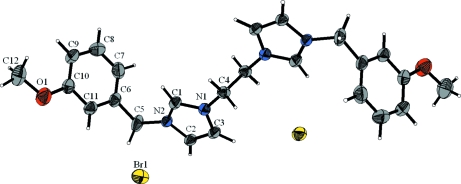
The structure of the title compound, showing 50% probability displacement ellipsoids for the non-hydrogen atoms. The H atoms are depicted as circles of arbitrary radius. The unlabelled atoms of the imidazolium cation are related to the labelled ones by 3/2 - x, 3/2 - y, -z; for the anion, the symmetry operation for Br1 is 1 - x + 1, y, 1/2 - z.

**Fig. 2.**
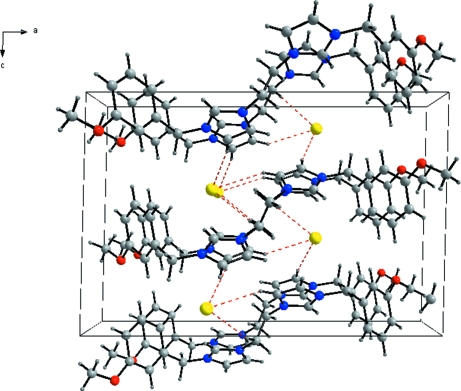
A view of the crystal packing along the b axis. Hydrogen bonds are shown as dashed lines.

## supplementary crystallographic information

### Comment

In the past decade, N-heterocyclic carbenes (NHCs) and their palladium complexes have attracted much interest due to their catalytic activities in C—C coupling reactions (Hillier *et al.*, 2002). The structure of 1,1'-bis(3-methoxybenzyl)-3,3'-methylenediimidazolium dibromide has already been reported (Lee & Chiu, 2004). The structures of other related bis(imidazolium) salts have also been reported (Cheng *et al.*, 2006; Lee *et al.*, 2007).

One of the common methods for the preparation of palladium NHC complexes is a one-pot reaction between an imidazolium salt and a palladium precursor in the presence of base (Lee *et al.*, 2004). By this method, we prepared a palladium bis(NHC) complex from the title compound. Here, we report the crystal structure of the title compound.

The structure of the title compound is shown in Fig. 1. The bis(imidazolium) dication is located on an inversion center, with the two imidazole rings parallel to each other. The imidazole and benzene rings make a dihedral angle of 77.25 (16)°. The bromide anions are involved in intermolecular hydrogen bonds of the type C—H···Br with the imidazolium cations, forming a three-dimensional hydrogen-bonded network (Fig. 2 and Table 1).

### Experimental

The compound was prepared according to the literature procedure (Lee *et al.*, 2004). Suitable crystals were obtained by slow diffusion of diethyl ether into a DMF solution of the compound at room temperature. The average dimensions of the colorless, rod-like crystals are about 0.35 x 0.20 x 0.20 mm.

### Refinement

All hydrogen atoms could have been located in the difference Fourier map; nevertheless, they were all positioned geometrically and refined as riding atoms, with C_aryl_—H = 0.93, C_methyl_ —H = 0.96, C_methylene_—H = 0.97 Å; *U*_iso_(H) = 1.5*U*_eq_(C) for the methyl H atoms and *U*_iso_(H) = 1.2*U*_eq_(C) for all the other H atoms.

### Crystal data

**Table:**

C_24_H_28_N_4_O_2_^2+^·2Br^−^	*F*(000) = 572
*M**_r_* = 564.32	*D*_x_ = 1.546 Mg m^−^^3^
Monoclinic, *P*2_1_/*c*	Mo *K*α radiation, λ = 0.71073 Å
Hall symbol: -P 2ybc	Cell parameters from 1952 reflections
*a* = 18.340 (6) Å	θ = 3.3–26.3°
*b* = 5.3566 (17) Å	µ = 3.37 mm^−^^1^
*c* = 12.340 (4) Å	*T* = 298 K
β = 91.491 (9)°	Rod, white
*V* = 1211.9 (7) Å^3^	0.35 × 0.20 × 0.15 mm
*Z* = 2	

### Data collection

**Table:**

Bruker SMART APEXII diffractometer	2609 independent reflections
Radiation source: fine-focus sealed tube	1838 reflections with *I* > 2σ
graphite	*R*_int_ = 0.050
ω scans	θ_max_ = 27.0°, θ_min_ = 3.3°
Absorption correction: multi-scan (SADABS; Sheldrick, 2003)	*h* = −14→23
*T*_min_ = 0.366, *T*_max_ = 0.600	*k* = −6→6
6855 measured reflections	*l* = −14→15

### Refinement

**Table:**

Refinement on *F*^2^	Primary atom site location: structure-invariant direct methods
Least-squares matrix: full	Secondary atom site location: difference Fourier map
*R*[*F*^2^ > 2σ(*F*^2^)] = 0.052	Hydrogen site location: difference Fourier map
*wR*(*F*^2^) = 0.143	H-atom parameters constrained
*S* = 1.00	*w* = 1/[σ^2^(*F*_o_^2^) + (0.0825*P*)^2^ + 0.0975*P*] where *P* = (*F*_o_^2^ + 2*F*_c_^2^)/3
2609 reflections	(Δ/σ)_max_ = 0.001
145 parameters	Δρ_max_ = 0.71 e Å^−^^3^
0 restraints	Δρ_min_ = −1.24 e Å^−^^3^

### Special details

**Table:**

Geometry. All e.s.d.'s (except the e.s.d. in the dihedral angle between two l.s. planes) are estimated using the full covariance matrix. The cell e.s.d.'s are taken into account individually in the estimation of e.s.d.'s in distances, angles and torsion angles; correlations between e.s.d.'s in cell parameters are only used when they are defined by crystal symmetry. An approximate (isotropic) treatment of cell e.s.d.'s is used for estimating e.s.d.'s involving l.s. planes.
Refinement. Refinement of *F*^2^ against ALL reflections. The weighted *R*-factor *wR* and goodness of fit *S* are based on *F*^2^, conventional *R*-factors *R* are based on *F*, with *F* set to zero for negative *F*^2^. The threshold expression of *F*^2^ > σ(*F*^2^) is used only for calculating *R*-factors(gt) *etc*. and is not relevant to the choice of reflections for refinement. *R*-factors based on *F*^2^ are statistically about twice as large as those based on *F*, and *R*- factors based on ALL data will be even larger.

### Fractional atomic coordinates and isotropic or equivalent isotropic displacement parameters (Å^2^)

**Table:**

	*x*	*y*	*z*	*U*_iso_*/*U*_eq_	
N1	0.43660 (17)	0.3916 (6)	0.1046 (3)	0.0292 (7)	
N2	0.33797 (15)	0.2134 (6)	0.1563 (2)	0.0272 (7)	
O1	0.09061 (18)	0.8336 (7)	0.1978 (3)	0.0551 (9)	
Br1	0.34564 (2)	0.59558 (9)	0.39315 (4)	0.0443 (2)	
C1	0.3639 (2)	0.4034 (7)	0.1010 (3)	0.0318 (9)	
H1A	0.3361	0.5252	0.0655	0.038*	
C2	0.3950 (2)	0.0735 (7)	0.1966 (3)	0.0305 (9)	
H2A	0.3917	−0.0702	0.2385	0.037*	
C3	0.4573 (2)	0.1845 (8)	0.1637 (3)	0.0331 (9)	
H3A	0.5048	0.1309	0.1783	0.040*	
C4	0.4858 (2)	0.5616 (7)	0.0499 (3)	0.0301 (9)	
H4A	0.5262	0.6056	0.0984	0.036*	
H4B	0.4600	0.7136	0.0299	0.036*	
C5	0.2612 (2)	0.1516 (9)	0.1779 (4)	0.0478 (13)	
H5A	0.2538	0.1573	0.2554	0.057*	
H5B	0.2511	−0.0173	0.1534	0.057*	
C6	0.2085 (2)	0.3286 (8)	0.1218 (4)	0.0353 (10)	
C7	0.1942 (2)	0.3068 (10)	0.0106 (4)	0.0504 (12)	
H7A	0.2172	0.1854	−0.0300	0.060*	
C8	0.1452 (3)	0.4690 (11)	−0.0375 (4)	0.0549 (13)	
H8A	0.1360	0.4578	−0.1119	0.066*	
C9	0.1094 (2)	0.6460 (9)	0.0203 (4)	0.0470 (12)	
H9A	0.0763	0.7530	−0.0142	0.056*	
C10	0.1230 (2)	0.6648 (8)	0.1315 (4)	0.0375 (10)	
C11	0.1735 (2)	0.5067 (9)	0.1817 (4)	0.0361 (9)	
H11A	0.1835	0.5211	0.2557	0.043*	
C12	0.0360 (3)	0.9930 (12)	0.1522 (5)	0.0641 (15)	
H12A	0.0182	1.1019	0.2073	0.096*	
H12B	−0.0035	0.8940	0.1231	0.096*	
H12C	0.0564	1.0909	0.0953	0.096*	

### Atomic displacement parameters (Å^2^)

**Table:**

	*U*^11^	*U*^22^	*U*^33^	*U*^12^	*U*^13^	*U*^23^
N1	0.0268 (16)	0.0313 (18)	0.0295 (17)	−0.0003 (13)	0.0001 (13)	0.0021 (14)
N2	0.0241 (16)	0.0276 (18)	0.0298 (17)	0.0021 (13)	−0.0011 (13)	0.0040 (14)
O1	0.053 (2)	0.053 (2)	0.060 (2)	0.0171 (16)	−0.0040 (16)	−0.0099 (17)
Br1	0.0467 (3)	0.0480 (3)	0.0380 (3)	−0.0161 (2)	−0.00102 (19)	0.0012 (2)
C1	0.030 (2)	0.030 (2)	0.036 (2)	0.0073 (16)	−0.0012 (16)	0.0039 (17)
C2	0.029 (2)	0.030 (2)	0.032 (2)	−0.0002 (16)	−0.0018 (16)	0.0090 (17)
C3	0.029 (2)	0.036 (2)	0.034 (2)	0.0047 (17)	−0.0043 (16)	0.0071 (18)
C4	0.031 (2)	0.031 (2)	0.028 (2)	−0.0029 (16)	−0.0002 (16)	0.0006 (16)
C5	0.021 (2)	0.051 (3)	0.071 (3)	−0.0005 (19)	0.004 (2)	0.024 (2)
C6	0.0225 (19)	0.037 (2)	0.047 (2)	−0.0039 (17)	−0.0012 (17)	0.0074 (19)
C7	0.038 (3)	0.059 (3)	0.055 (3)	0.008 (2)	0.007 (2)	−0.008 (3)
C8	0.054 (3)	0.075 (4)	0.036 (3)	0.009 (3)	−0.006 (2)	−0.002 (2)
C9	0.036 (2)	0.055 (3)	0.049 (3)	0.011 (2)	−0.006 (2)	0.010 (2)
C10	0.029 (2)	0.037 (2)	0.046 (3)	−0.0011 (18)	0.0003 (18)	−0.0017 (19)
C11	0.028 (2)	0.043 (2)	0.038 (2)	−0.0042 (19)	−0.0045 (17)	0.003 (2)
C12	0.052 (3)	0.056 (3)	0.084 (4)	0.020 (3)	0.003 (3)	−0.006 (3)

### Geometric parameters (Å, °)

**Table:**

N1—C1	1.334 (5)	C5—H5A	0.9700
N1—C3	1.375 (5)	C5—H5B	0.9700
N1—C4	1.460 (5)	C6—C11	1.376 (6)
N2—C1	1.321 (5)	C6—C7	1.396 (6)
N2—C2	1.369 (5)	C7—C8	1.374 (7)
N2—C5	1.477 (5)	C7—H7A	0.9300
O1—C10	1.366 (5)	C8—C9	1.365 (7)
O1—C12	1.422 (6)	C8—H8A	0.9300
C1—H1A	0.9300	C9—C10	1.391 (6)
C2—C3	1.360 (6)	C9—H9A	0.9300
C2—H2A	0.9300	C10—C11	1.389 (6)
C3—H3A	0.9300	C11—H11A	0.9300
C4—C4^i^	1.502 (7)	C12—H12A	0.9600
C4—H4A	0.9700	C12—H12B	0.9600
C4—H4B	0.9700	C12—H12C	0.9600
C5—C6	1.509 (6)		
			
C1—N1—C3	108.6 (3)	C6—C5—H5B	109.2
C1—N1—C4	125.7 (3)	H5A—C5—H5B	107.9
C3—N1—C4	125.6 (3)	C11—C6—C7	120.5 (4)
C1—N2—C2	109.1 (3)	C11—C6—C5	119.5 (4)
C1—N2—C5	128.6 (3)	C7—C6—C5	120.0 (4)
C2—N2—C5	122.3 (3)	C8—C7—C6	118.5 (5)
C10—O1—C12	118.2 (4)	C8—C7—H7A	120.8
N2—C1—N1	108.6 (3)	C6—C7—H7A	120.8
N2—C1—H1A	125.7	C9—C8—C7	122.1 (5)
N1—C1—H1A	125.7	C9—C8—H8A	119.0
C3—C2—N2	107.0 (3)	C7—C8—H8A	119.0
C3—C2—H2A	126.5	C8—C9—C10	119.4 (4)
N2—C2—H2A	126.5	C8—C9—H9A	120.3
C2—C3—N1	106.7 (3)	C10—C9—H9A	120.3
C2—C3—H3A	126.6	O1—C10—C11	115.5 (4)
N1—C3—H3A	126.6	O1—C10—C9	124.7 (4)
N1—C4—C4^i^	109.6 (4)	C11—C10—C9	119.7 (4)
N1—C4—H4A	109.7	C6—C11—C10	119.9 (4)
C4^i^—C4—H4A	109.7	C6—C11—H11A	120.1
N1—C4—H4B	109.7	C10—C11—H11A	120.1
C4^i^—C4—H4B	109.7	O1—C12—H12A	109.5
H4A—C4—H4B	108.2	O1—C12—H12B	109.5
N2—C5—C6	112.3 (3)	H12A—C12—H12B	109.5
N2—C5—H5A	109.2	O1—C12—H12C	109.5
C6—C5—H5A	109.2	H12A—C12—H12C	109.5
N2—C5—H5B	109.2	H12B—C12—H12C	109.5

Symmetry codes: (i) −*x*+1, −*y*+1, −*z*.

### Hydrogen-bond geometry (Å, °)

**Table:**

*D*—H···*A*	*D*—H	H···*A*	*D*···*A*	*D*—H···*A*
C2—H2A···Br1^ii^	0.93	2.77	3.657 (4)	161
C3—H3A···Br1^iii^	0.93	2.91	3.729 (4)	148
C4—H4B···Br1^iv^	0.97	2.85	3.669 (4)	143

Symmetry codes: (ii) *x*, *y*−1, *z*; (iii) −*x*+1, *y*−1/2, −*z*+1/2; (iv) *x*, −*y*+3/2, *z*−1/2.
